# Supplementary material for: Potential present and future distributions of the genus Atta of Mexico
Source: PLoS One. 2023 Sep 26;18(9):e0292072. doi: 10.1371/journal.pone.0292072 (PMC10522027; doi:10.1371/journal.pone.0292072)
Supplement: S2 Table — Bioclimatic variables (names) used as predictors in the species distribution models of three Mexican leaf-cutting ant species (A. cephalotes, A. mexicana, and A. texana). (DOCX) [file pone.0292072.s003.docx]

**Table 1**. Bioclimatic variables (names) used as predictors in the species distribution models of three Mexican leaf-cutting ant species (*A. cephalotes*, *A*. *mexicana*, and *A*. *texana*).

| **Variable** | **Name** | **Species** |
| --- | --- | --- |
| Bio 2 | Mean Diurnal Range | *A. cephalotes*, *A*. *mexicana, A*. *texana* |
| Bio 3 | Isothermality | *A*. *mexicana* |
| Bio 4 | Temperature Seasonality | *A*. *cephalotes* |
| Bio 5 | Max Temperature of Warmest Month | *A*. *mexicana* |
| Bio 6 | Min Temperature of Coldest Month | *A*. *texana* |
| Bio 8 | Mean Temperature of Wettest Quarter | *A*. *cephalotes*, *A*. *mexicana, A*. *texana* |
| Bio 9 | Mean Temperature of Driest Quarter | *A*. *texana* |
| Bio 13 | Precipitation of Wettest Month | *A*. *cephalotes*, *A*. *mexicana, A*. *texana* |
| Bio 14 | Precipitation of Driest Month | *A*. *cephalotes, A*. *mexicana* |
| Bio 15 | Precipitation Seasonality (Coefficient of Variation) | *A*. *cephalotes*, *A*. *mexicana, A*. *texana* |
| Bio 18 | Precipitation of Warmest Quarter | *A*. *cephalotes*, *A*. *mexicana, A*. *texana* |
| Bio 19 | Precipitation of Coldest Quarter | *A*. *mexicana* |
