# Supplementary material for: Potential present and future distributions of the genus Atta of Mexico
Source: PLoS One. 2023 Sep 26;18(9):e0292072. doi: 10.1371/journal.pone.0292072 (PMC10522027; doi:10.1371/journal.pone.0292072)
Supplement: Attachment — Submitted filename: rebuttal letter Plos Atta 2.docx [file pone.0292072.s004.docx]

**Dear Dr. Volker Nehring**

Academic Editor of PLOS ONE,

I hope this message finds you well. We have made the necessary corrections to our article "Potential present and future distributions of the genus *Atta* of Mexico" previously submitted to your journal. After receiving the comments and suggestions from the expert reviewers, we have dedicated time and effort to address each of the points raised and improve the overall quality of the article. We are pleased to inform you that we have completed the revisions per the guidelines. We want to summarize the key modifications made in response to the received comments:

1. Additional information was incorporated regarding the ecological roles of Atta ants and their significance as pests. Specifically, we emphasized their voracious foraging behavior, leading to defoliation across various plant species and the consequent economic impacts.

2. A change in the author order was effected due to the substantial contribution of the corresponding author in species distribution modeling and addressing review comments.

3. Detailed insight was provided on how the models account for spatial autocorrelation. We highlighted using algorithms like MaxEnt, SVM, and ANN, which adeptly handle spatial autocorrelation.

4. The table was titled "Ecoregions per Species Based on Known Distributions," reflecting the basis of terrestrial ecoregions and the selection of ecoregions where the distribution of each species was known.

5. We decided to remove Cohen's Kappa coefficient from the analysis as it was deemed potentially redundant alongside other metrics, in line with the reviewer's observation, aiming to streamline the data presentation.

These changes were implemented to enhance the manuscript's clarity and quality, and we hope they adequately address the reviewer's concerns. We are grateful to the reviewers for their valuable comments and suggestions, as they have significantly helped us enhance the quality and rigor of our article. These modifications strengthen the robustness of our research and increase its relevance within the scientific community. We have attached the file with the implemented corrections for your review. Upon evaluating the revisions, we hope our article will be deemed suitable for publication in PLOS ONE. Thank you sincerely for your time and consideration. We look forward to your response and remain available for any further inquiries that may arise.

Yours sincerely,

Jorge Antonio Gómez Díaz

Instituto de Investigaciones Biológicas, Universidad Veracruzana

jorggomez@uv.mx

**Detailed response**

**Editor**

1. *For me it wasn't entirely clear if the distribution data you used functions as presence only data or if the absence of a species from locations also factored in.*

R = Thank you for your comments. We understand your concern regarding the nature of the distribution data used in our study. True absence data, especially for mobile species like ants, is rare in ecology. Instead, we employed pseudo-absences, a common approach in species distribution models (SDMs) when true absence data is lacking (Barbet‐Massin et al. 2012). The algorithms we used, specifically MaxEnt, SVM, and ANN, operate using these pseudo-absences. They require background or pseudo-absence data, even if occasionally mislabeled as presence-only methods. To clarify, our study did not utilize confirmed absence data for the species studied but relied on generated pseudo-absences in line with standard practices in the field. We included an explanation of this in the methodology section (L 137-140): “Our study employed pseudo-absence data, as is standard in species distribution models (SDMs) when true absence data is unavailable [23]. We used algorithms, specifically MaxEnt, SVM, and ANN, which necessitate pseudo-absences, adhering to recognized ecological modeling practices.” We hope this provides clarity, and we are willing to make necessary adjustments to the manuscript to reflect this more explicitly.

2. *I did not understand from what area the partial ROC was calculated. In the text a 5% threshold is mentioned, but 5% of what does that refer to?*

R = The 5% mentioned refers to the allowable omission error for the evaluated models (Peterson et al. 2008). In other words, it sets a threshold whereby models that omit known presence points more than 5% of the time are considered less reliable. This threshold was chosen based on the quality and error characteristics of the occurrence data used. Essentially, models with an omission rate (OR) ≤ 0.05 were showcased as they err by omitting known points of presence less than or equal to 5% of the time. This is critical for niche modeling, where models that miss known presence points are deemed more flawed than those that might over-predict areas. We included this in the methodology section (L 147-150): “The referenced 5% pertains to the allowable omission error in the models assessed [25]. Specifically, models that omit known presence points more than 5% of the time are deemed less reliable. This threshold was chosen based on the error characteristics of the occurrence data used.”

3. *Do the models take care of potential autocorrelation?*

R = In response to concerns regarding spatial autocorrelation (SAC), our methodology was meticulously designed to counteract its effects. Firstly, we ensured data accuracy and minimization of spatial clustering by using the `clean_coordinates` function from the CoordinateCleaner R package, retaining only one occurrence per km^2^. Although SAC can infringe upon the 'independent errors' assumption of SDMs, we opted for algorithms inherently robust against spatial autocorrelation. Specifically, we employed MaxEnt, SVM, and ANN. These algorithms are adept at handling SAC and benefit from a comprehensive set of occurrence points, ensuring a thorough exploration of the environmental space and thereby bolstering their robustness.

**Reviewer #1**

1. *In particular, I only consider that in the Introduction section, the authors could mention some basic aspects of the ecological functions of* Atta *and their role as pests.*

R = Thank you for your suggestion. We've incorporated information about the ecological functions of *Atta* and their role as pests in the Introduction section (L 55-61): “*Atta* ants, often called leafcutter ants, play essential ecological roles in ecosystems, particularly in nutrient cycling and soil aeration [6]. Their intricate underground colonies contribute to soil turnover [6]. However, they are also renowned as significant agricultural pests, with their voracious foraging behavior leading to the defoliation of a wide range of plant species, causing notable economic impacts in affected regions [7]. *Atta* species utilize all the leaves they cut to cultivate fungi in their underground chambers [6].”

2. *L3: The order of the authors changed, which made me curious.*

R = In response to the reviewer's query regarding the change in the order of authors: The corresponding author, now the first author, was primarily responsible for executing all aspects of species distribution modeling and addressing all comments from the initial review. Given these significant contributions and leadership in revisions, all co-authors unanimously agreed that it would be appropriate for them to be listed as the first author.

3. *L62: The authors could briefly mention the ecological functions of Atta to support line 68. In addition, I think that the role of these ants as pests could also be highlighted, which enhances the importance of this study.*

R = As addressed in our first response, we have incorporated information detailing the ecological functions of *Atta* (L 55-61). Moreover, further text has been added to emphasize their role as pests, which underscores the significance of this study (L 74-77): “Given Atta ants' unique behavior of cultivating fungi using cut leaves, the challenges they pose to agriculture cannot be understated. Recognizing their ecological significance and impact on agriculture, this study seeks insights crucial for effective colony control and strategic management, as reviewed by [6].” Thank you for pointing out the importance of highlighting these aspects.

4. *L108: I suggest mentioning the ecoregions for each species.*

R = Since many ecoregions exist, we have included supplementary information (S2 Table).

5. *L200 (Figure 2): I believe that the names of the axes in the figure should only indicate PC1, PC2, and PC3, that is, eliminate “components$”.*

R = Done.

6. *L305: five (letter) or 5 (number)?*

R = We have changed to 5 (number).

**Reviewer #2**

1. *Line 25 - "minimum" instead of "min"*

R = Done.

2. *Line 35 - replace "in the face of" with "because of" or "due to"*

R = Done.

3. *Line 56 ("The relationship between...") - this is an opinion but I suppose it's OK in the introductory context*

R = We have changed the sentence, and it is not anymore an opinion (L 62-63): “The relationship between *Atta* ants and fungi is a well-documented symbiotic interaction.”

4. *Line 79 - "change" instead of "change's"*

R= Done.

5. *Line 87 - "Facility" instead of "Fund"*

R = Done.

6. *Line 128 - I think "ensemble" should be lower-case here*

R= Done

7. *Line 145 - "first three" instead of "three first"*

R= Done

8. *Line 160 - replace "evidencing the" with "suggesting"*

R = Done.

9. *Line 166 - "Minimum" instead of "Min"*

R = Done.

10. *Line 173 (Table 2) - I don't love Cohen's Kappa but I understand that it's one of evaluation metrics in SSDM. It doesn't really tell you anything not already captured by other metrics. In short, the ensemble for A. mexicana was the least 'successful' of the three (but still decent by most metrics). I'm not surprised given that A. mexicana has a rather 'skinny' ellipsoid in Fig 2.*

R = Thank you for pointing out the potential redundancy of Cohen's Kappa in the context of other metrics presented. While it's commonly used in species distribution modeling, its value might overlap with other metrics. To streamline the table and provide a clearer representation of the model's success, we have removed it from Table 2. We appreciate your keen observation and suggestion.

11. *Line 183 - italicize "Atta"*

R = Done.

12. *Lines 189-198 - The mix of lower-case and upper-case variable names in this paragraph is a little jarring.*

R = We have changed and homogenized the terms to lowercase when necessary.

13. *Line 250 - consider replacing "initial" with "first"*

R= Done.

14. *Line 301 - "the Americas" instead of "America"*

R = Done.

15. *Line 305 - "5" instead of "five" (since it refers to a specific degrees C value).*

R = Done

**References**

Barbet‐Massin, M., Jiguet, F., Albert, C. H., & Thuiller, W. (2012). Selecting pseudo‐absences for species distribution models: How, where and how many? Methods in ecology and evolution, 3(2), 327-338.

Peterson, A. T., Papeş, M., & Soberón, J. (2008). Rethinking receiver operating characteristic analysis applications in ecological niche modeling. Ecological modelling, 213(1), 63-72.
